# Evaluation of risk factors for sleep‐disordered breathing in dogs

**DOI:** 10.1111/jvim.17019

**Published:** 2024-02-15

**Authors:** Iida Niinikoski, Sari‐Leena Himanen, Mirja Tenhunen, Mimma Aromaa, Liisa Lilja‐Maula, Minna M. Rajamäki

**Affiliations:** ^1^ Department of Equine and Small Animal Medicine University of Helsinki Helsinki Finland; ^2^ Faculty of Medicine and Health Technology Tampere University Tampere Finland; ^3^ Department of Clinical Neurophysiology Tampere University Hospital, Wellbeing Services County of Pirkanmaa Tampere Finland; ^4^ Department of Medical Physics Tampere University Hospital, Wellbeing Services County of Pirkanmaa Tampere Finland

**Keywords:** brachycephalic obstructive airway syndrome, obstructive respiratory event index, obstructive sleep apnea, sleep‐disordered breathing

## Abstract

**Background:**

Brachycephalic dogs display sleep‐disordered breathing (SDB). The risk factors for SDB remain unknown.

**Objectives:**

To identify risk factors for SDB. We hypothesized that brachycephaly, increasing severity of brachycephalic obstructive airway syndrome (BOAS), excess weight, and aging predispose to SDB.

**Animals:**

Sixty‐three privately owned pet dogs were prospectively recruited: 28 brachycephalic and 35 normocephalic (mesaticephalic or dolicocephalic) dogs.

**Methods:**

Prospective observational cross‐sectional study with convenience sampling. Recording with the neckband was done over 1 night at each dog's home. The primary outcome measure was the obstructive respiratory event index (OREI). Body condition score (BCS) was assessed, and BOAS severity was graded for brachycephalic dogs.

**Results:**

Brachycephaly was a significant risk factor for high OREI value (ratio of the geometric means 5.6, 95% confidence interval [CI] 3.2‐9.9; *P* < .001) but aging was not (1.1, 95% CI 1.0‐1.2; *P* = .2). Excess weight, defined as a BCS of over 5/9, (3.5, 95% CI 1.8‐6.7; *P* < .001) was a significant risk factor. In brachycephalic dogs, BOAS‐positive class (moderate or severe BOAS signs) was a significant risk factor (2.5, 95% CI 1.1‐5.6; *P* = .03).

**Conclusions and Clinical Importance:**

Brachycephaly decreases welfare in a multitude of ways, including disrupting sleep. Brachycephaly, increasing severity of BOAS and excess weight are risk factors for obstructive SDB.

**Abbreviations**
BCS: body condition score
BOAS: brachycephalic obstructive airway syndrome
CI: confidence interval
OREI: obstructive respiratory event index
OSA: obstructive sleep apnea
REM: rapid eye movement
SBD: sleep‐disordered breathing

## 1 INTRODUCTION

Obstructive sleep‐disordered breathing (SDB) occurs in brachycephalic dogs.^1^, ^2^, ^3^, ^4^ In people SDB is an important health problem and in adults can be categorized into obstructive sleep apnea (OSA), central sleep apnea (CSA), sleep‐related hypoventilation, and sleep‐related hypoxemia.^5^ In brachycephalic dogs SDB seems to mirror human OSA, where hypopneas, partial blockages of airflow, and apneas, cessations of breathing, occur because of partial or complete obstruction of the upper airways.^5^ SDB has been characterized in modest groups of dogs.^1^, ^2^, ^3^, ^4^ Brachycephalic obstructive airway syndrome (BOAS) results from an excessively short anatomical skull structure and leads to a varying degree of upper airway obstruction.^6^, ^7^, ^8^, ^9^ Besides BOAS, upper airway obstruction has also been shown to result from airway lymphoedema leading to increased airflow resistance in normocephalic Norwich Terriers.^10^, ^11^, ^12^ Owner‐perceived signs of abnormal breathing during sleep and their effect on sleeping habits of dogs with BOAS include sleeping chin elevated or in a sitting position, snoring, apneic episodes during sleep, and difficulty to sleep at all.^8^, ^13^, ^14^, ^15^

Diagnostic methods for SDB evaluation in dogs include polysomnography^1^ and whole‐body barometric plethysmography,^3^ and recently, we reported successful use of a portable neckband system at the dog's home environment.^4^ The neckband system is used for 1 night and it provides the rate of obstructive SDB events.^4^

Despite knowledge on the risk factors for OSA in humans, there is no information, excluding brachycephaly, on aspects predisposing dogs to SDB. SDB has not been described in normocephalic dogs. In humans, features resembling the anatomical aspects seen in brachycephalic dogs, including decreased upper airway size combined with substantial upper airway soft tissue volume and other abnormalities in craniofacial and upper airway anatomy, predispose to OSA.^16^, ^17^, ^18^, ^19^, ^20^ The primary risk factor for OSA is obesity,^21^ and weight loss is an effective treatment form.^22^ In people, the prevalence of OSA increases consistently with age.^23^, ^24^, ^25^ There is later onset for women,^23^ where hormonal status, that is, lower progesterone levels after menopause, depress upper airway dilator muscle activity.^26^, ^27^

The objective of this study was to identify risk factors for obstructive SDB in dogs. We hypothesized that brachycephaly, increasing severity of BOAS, increasing age, and increasing obesity predispose to SDB.

## 2 MATERIALS AND METHODS

### 2.1 Study group

The study protocol was approved by the Committee of Experimental Animals of Southern Finland (ESAVI/10906/04.10.07/2017, ESAVI/34278/15.11.21/2021) and by the University of Helsinki Viikki Campus Research Ethics Committee (13/2020, 11/2021).

This prospective, observational cross‐sectional study with convenience sampling was performed at the Veterinary Teaching Hospital, University of Helsinki, Finland, and the Kaarina Veterinary Clinic, Kaarina, Finland, between October 2020 and February 2023. All animals were privately owned pet dogs. The owners signed an informed consent form before participation.

We developed an online questionnaire based on previous questionnaires for sleep dysfunction in dogs and OSA questionnaires for humans.^14^, ^28^, ^29^ The questionnaire was designed iteratively among the authors and tested with dog owners of a non‐veterinary background. Replies were collected by promoting it in the Finnish Kennel Club magazine and social media platforms and through breed clubs. The questionnaire was managed using REDCap electronic data capture tools hosted at the University of Helsinki.^30^, ^31^ Briefly, questions were grouped into 6 parts: demographics, medical history, sleeping customs, signs of sleep‐disturbed breathing (restless sleep, snoring, sleeping sitting up, sleeping with toy in mouth, sleeping with head hanging off the bed, waking up gasping, apneic episodes), sleepiness, and signs of other sleep disorders. The questionnaire is presented in Data S1, Supporting Information.

Dogs were selected for sleep recordings from the questionnaire replies. The inclusion criteria were age of over 1‐year, minimum weight of 4 kg, and for intact female dogs, anestrus. Pregnant and lactating dogs and dogs with medications affecting sleep or breathing during sleep, such as tricyclic antidepressants, gabapentin and ondansetron, or gastroesophageal reflux requiring treatment, were excluded. Dogs were not selected randomly, as brachycephalic dogs and both brachycephalic and normocephalic dogs with owner‐perceived signs of abnormal breathing during sleep were emphasized. Further normocephalic dogs with no owner‐perceived signs of abnormal breathing during sleep were also selected. Some of both the normocephalic (n = 12/63) and brachycephalic (n = 12/63) dogs have participated in a previous pilot study, where only their obstructive respiratory event index (OREI) results were reported.^4^

### 2.2 Study protocol for dogs with sleep recordings

Most dogs with sleep recordings attended a study visit, during which a physical examination was performed, and blood samples including hematology and biochemistry for health verification obtained. Body condition score (BCS) was recorded on a 9‐point scale. For the brachycephalic dogs, severity grading of BOAS was performed during the study visit or within 6 months of the sleep recording as previously presented.^9^, ^32^ Briefly, brachycephalic dogs were graded as having no (grade 0), mild (grade 1), moderate (grade 2), or severe (grade 3) BOAS signs. Based on this grading, the dogs were classified as BOAS‐negative (BOAS −; grades 0 and 1) or BOAS‐positive (BOAS +; grades 2 and 3) classes.

Breathing during sleep was assessed from portable neckband system recordings. The portable neckband system (Nukute Ltd., Oulu, Finland) was used in conjunction with a protective cover to ensure the device did not fall off the dog's neck. Otherwise, recordings were performed during 1 night at home under the owner's supervision as previously described.^4^ To ensure sufficient rapid eye movement (REM) sleep, the minimum duration of sleep recording was 2 hours. Briefly, the neckband system consists of a c‐shaped neckband device, a tablet, and a pulse oximeter, which was not in use in this study (Berry BM2000D, Shanghai Berry Electronic Technology Co. Ltd., Shanghai, China). The neckband includes a piezoelectric microphone for tracheal sounds, an ambient microphone, and a gyroscope for data on position and movement. The audio was recorded with a sampling rate of 16 000 Hz and saved as 2‐channel 16‐bit integers. Breathing‐related signals were acquired from tracheal sound recordings and respiratory rate signal using audio. Gyroscope data were saved with 10 Hz sampling rate as 32‐bit integers. A picture of the neckband and protective cover is presented in Data S2, Supporting Information.

The owner placed the neckband and cover on the dog before going to bed and removed it after waking in the morning. This was defined as the duration of recording. Data were analyzed by an experienced sleep researcher (SLH) via the manufacturer's analysis software. As previously, the apnea and hypopnea events were scored manually per children's guidelines.^33^ The obstructive respiratory event results were summarized as OREI value, describing the number of obstructive apnea and hypopnea events per monitoring time.^4^ Additionally, the percentage of snoring, as time spent snoring of recorded time, was manually scored, and confirmed by listening.

### 2.3 Statistical methods

Continuous data were assessed for normality with the Shapiro‐Wilk test. Normally distributed data are presented as mean ± SD and non‐parametric data as median with range and/or interquartile range. The dogs were grouped into brachycephalic dogs and normocephalic dogs.

The potential risk factors (gender, neuter status, brachycephaly, age, excess weight defined as a BCS of over 5/9, and BOAS class) for high OREI value were first assessed separately with univariable analysis (1‐way analysis of variance or linear regression depending on the qualities of the risk factor). OREI value was log‐transformed for the analysis to comply with parametric modeling requirements. For interpretation, the estimates for the differences were backtransformed to original scale. The presented estimates are ratios between the groups, not absolute differences.

If more than 1 risk factor was significant in the univariable analysis, they were modeled together.

Statistical analyses were done using SAS System for Windows, version 9.4 (SAS Institute Inc., Cary, North Carolina, USA) and GraphPad Prism for Macintosh, version 9.3.0 (GraphPad Software, San Diego, CA, USA). *P*‐values < .05 were considered statistically significant.

## 3 RESULTS

### 3.1 Study group

**FIGURE 1:**
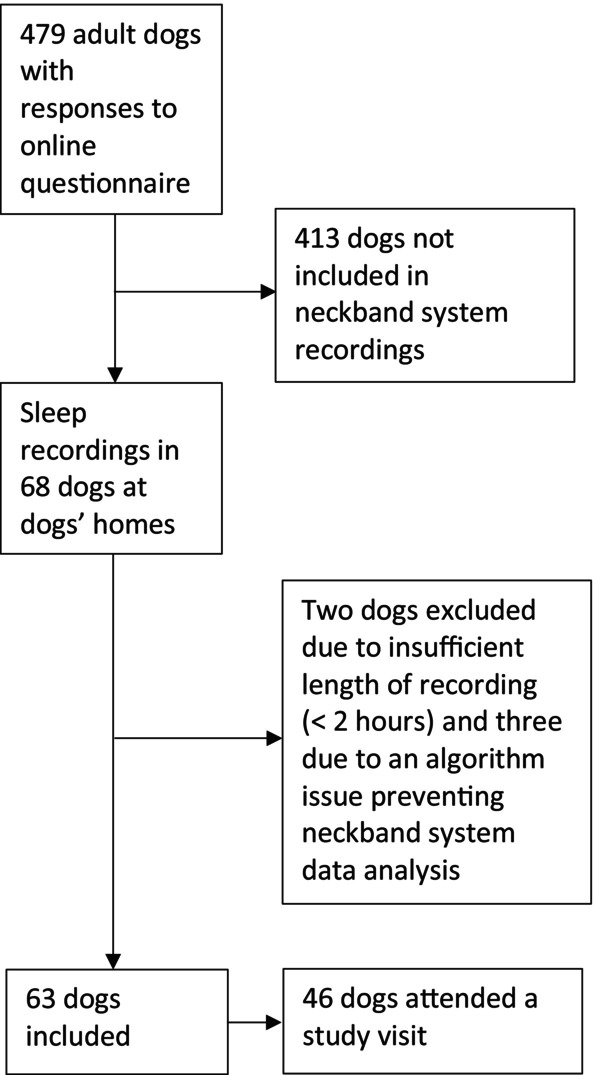
Flow chart of the selection process for sleep recordings.

Sixty‐eight adult pet dogs, housed indoors, were recruited from the online questionnaire replies for sleep recordings (Figure 1). The breed distribution for all dogs with successful sleep recordings is shown in Table 1. Of the 63 dogs with adequate sleep recordings, 28 were brachycephalic and 35 normocephalic. Twenty‐eight were female (13 intact and 15 neutered) and 35 were male (22 intact and 13 neutered). Median age was 5.8 years (range 1.4‐12.7 years). Median weight was 12.4 kg (range 4.7‐57.0 kg). Forty‐six dogs attended a study visit. Of these, 14 had a BCS of 4/9, 20 5/9, 9 6/9, 2 7/9 and 1 9/9, that is, 12 dogs had excess weight, defined as a BCS of over 5/9. Five brachycephalic dogs (5/19, 26%) and 7 normocephalic dogs (7/27, 26%) had a BCS of over 5/9. Nineteen brachycephalic dogs attending a study visit were graded for BOAS severity. Ten dogs were BOAS negative (6 grade 0 with no signs of BOAS and 4 grade 1 with mild signs of BOAS) and 9 were BOAS positive (4 grade 2 with moderate signs of BOAS and 5 grade 3 with severe signs of BOAS). BOAS positive dogs included 5 French Bulldogs, 2 Pugs, 1 Cavalier King Charles Spaniel, and 1 English Bulldog. One Bullmastiff, 1 French Bulldog, and 1 Pug had previously had BOAS surgery.

**TABLE 1:** Breed distribution and presence of owner‐perceived signs of sleep‐disordered breathing (SDB) in all 63 dogs with sleep recordings.

Normocephalic breeds	n = 35	Owner‐perceived signs of SDB	Brachycephalic breeds	n = 28	Owner‐perceived signs of SDB
Labrador Retriever	10	5/10	French Bulldog	10	7/10
Jack Russell Terrier	3	2/3	CKCS	8	3/8
Parson Jack Russell Terrier	3	0/3			
Bassett Fauve de Bretagne	2	0/2			
Border Collie	2	1/2			
Golden Retriever	2	0/2	Pug	4	4/4
Lancashire Heeler	2	1/2			
Miniature Schnauzer	2	2/2			
Norwich terrier	2	1/2			
Irish Setter	1	0/1	Boston Terrier	1	1/1
Kleinspitz	1	1/1	Boxer	1	0/1
Lapponian Herder	1	0/1	Bullmastiff	1	1/1
Mixed breed	1	1/1	English Bulldog	1	1/1
Spanish Water Dog	1	0/1	Petit Brabancon	1	1/1
Wales Terrier	1	0/1	Staffordshire Terrier	1	1/1
Whippet	1	0/1			
All		14/35 (40%)	All		19/28 (67%)

Abbreviation: CKCS, Cavalier King Charles Spaniel.

### 3.2 Signs of SDB

**FIGURE 2:**
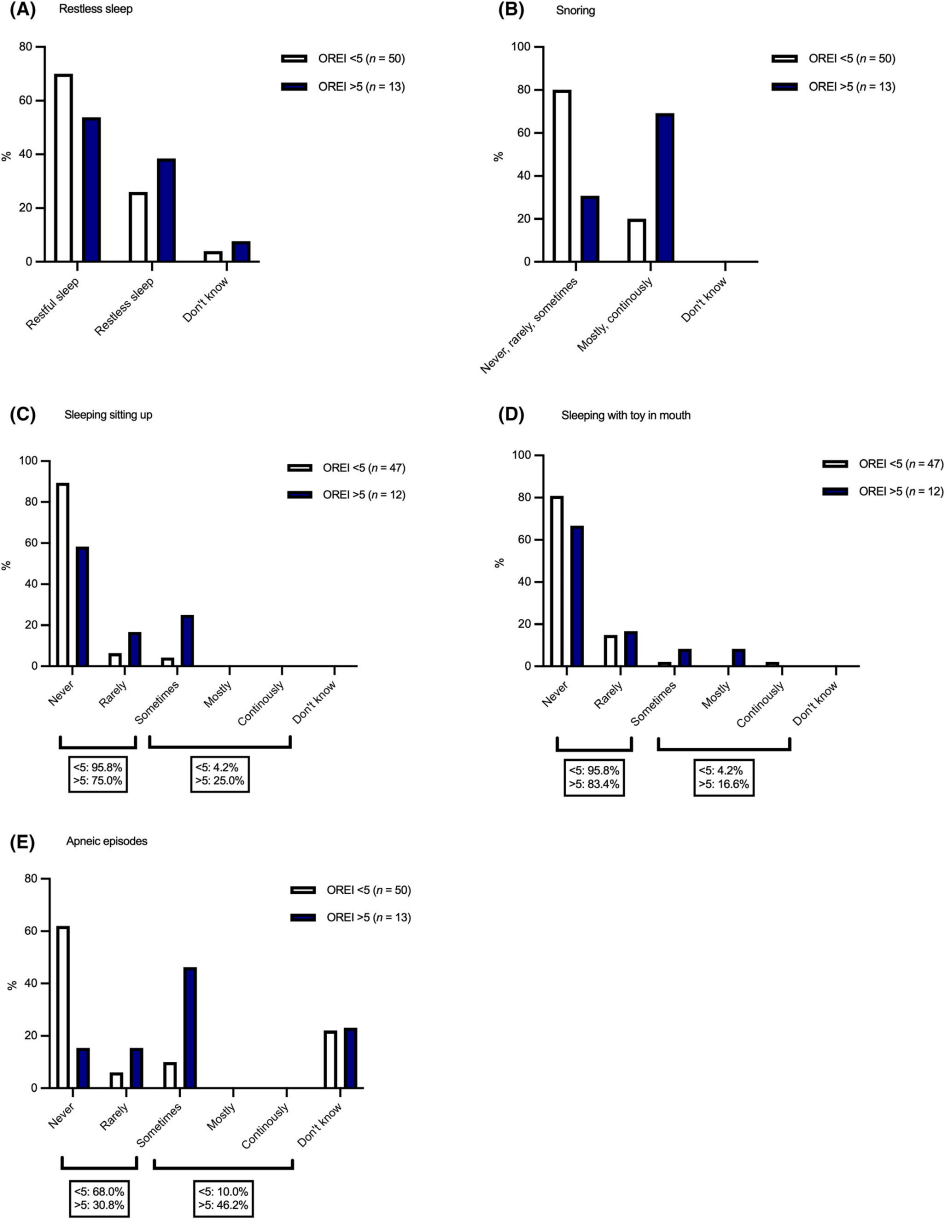
Occurrence of owner‐perceived signs of sleep‐disordered breathing, including restless sleep (A), snoring during sleep (B), sleeping sitting up (C), sleeping with toy in mouth (D), and apneic episodes during sleep (E), in dogs with a lower obstructive respiratory event index (OREI) of below 5 and higher OREI of over 5.

The most descriptive owner‐perceived signs depicting SDB were restless sleep, snoring, sleeping sitting up, sleeping with toy in mouth and apneic episodes during sleep (Figure 2A‐E). A cut‐off value of 5 was used for OREI, as under 5 apnea or hypopnea events per hour of sleep are considered normal in adult humans.^5^

**FIGURE 3:**
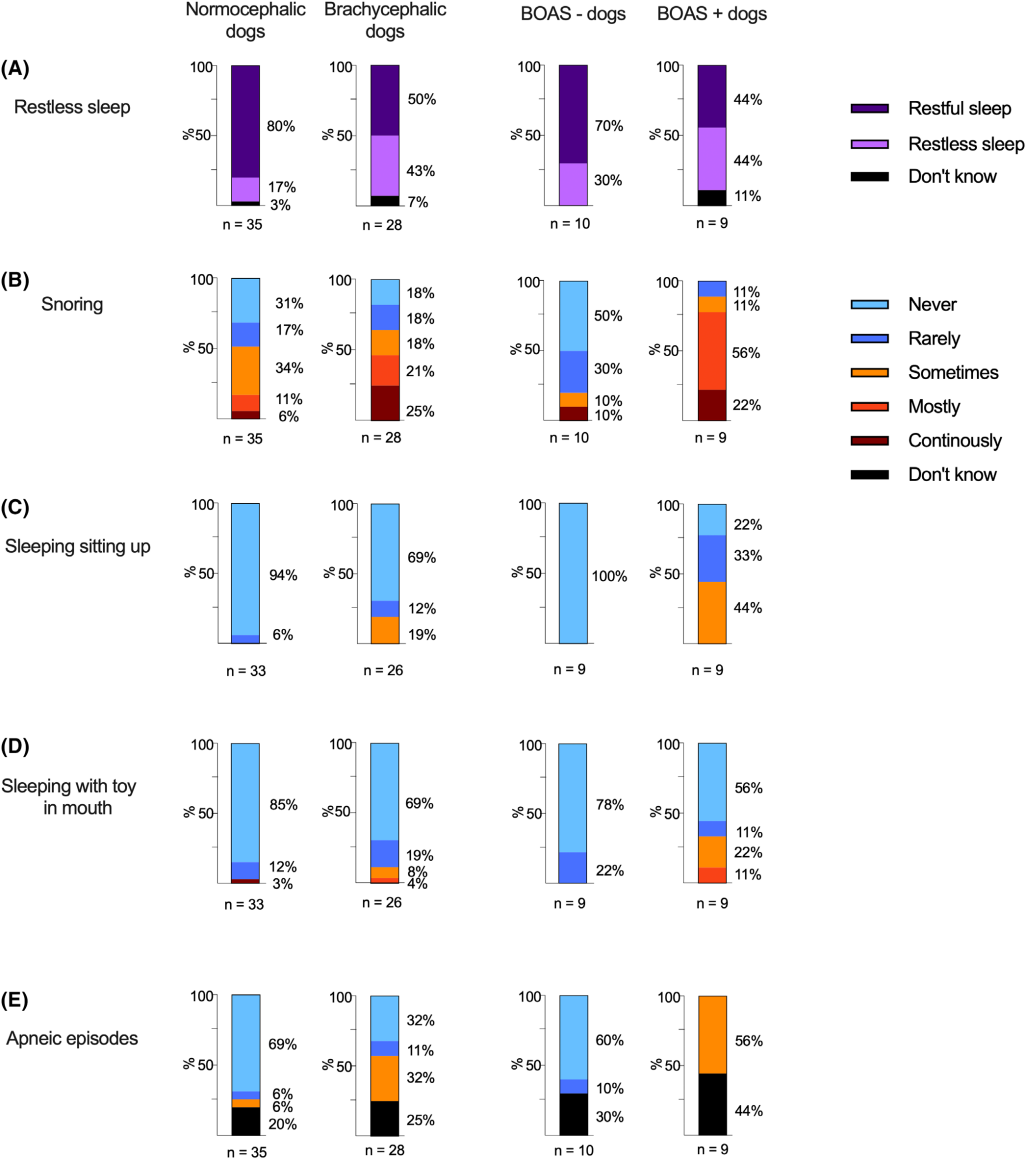
Occurrence of owner‐perceived signs of sleep‐disordered breathing, including restless sleep (A), snoring (B), sleeping sitting up (C), sleeping with toy in mouth (D), and apneic episodes during sleep (E), in normocephalic dogs and brachycephalic dogs, and in brachycephalic dogs with no or mild signs of brachycephalic obstructive airway syndrome (BOAS −) and brachycephalic dogs with moderate or severe signs of BOAS (BOAS +).

Occurrence of owner‐perceived signs of SDB (restless sleep, snoring, sleeping sitting up, sleeping with toy in mouth, and apneic episodes during sleep) in normocephalic dogs and brachycephalic dogs, and in BOAS − and BOAS + dogs are presented in Figure 3A‐E.

**FIGURE 4:**
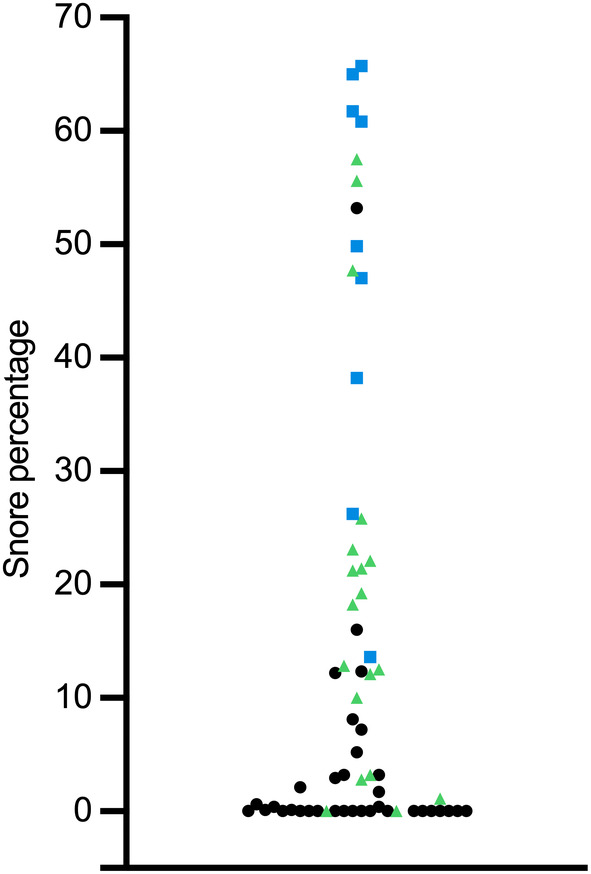
Scatter plot of snore percentage, as time spent snoring of total recording time, in all dogs with sleep recordings. Black data points are normocephalic dogs and green triangles and blue squares are brachycephalic dogs. Blue squares denote dogs with moderate or severe brachycephalic obstructive airway syndrome signs (BOAS positive class).

In all dogs, the median snore percentage was 3.2 (IQR 0.0‐21.4). In dogs with OREI over 5, the median snore percentage was 23.1 (IQR 13.2‐48.4) and in dogs with OREI under 5 the median was 0.85 (IQR 0.0‐12.3). For brachycephalic dogs, median snore percentage was 21.8 (IQR 12.2‐49.3) and normocephalic dogs 0.0 (IQR 0.0‐3.2; Figure 4).

### 3.3 OREI values

**FIGURE 5:**
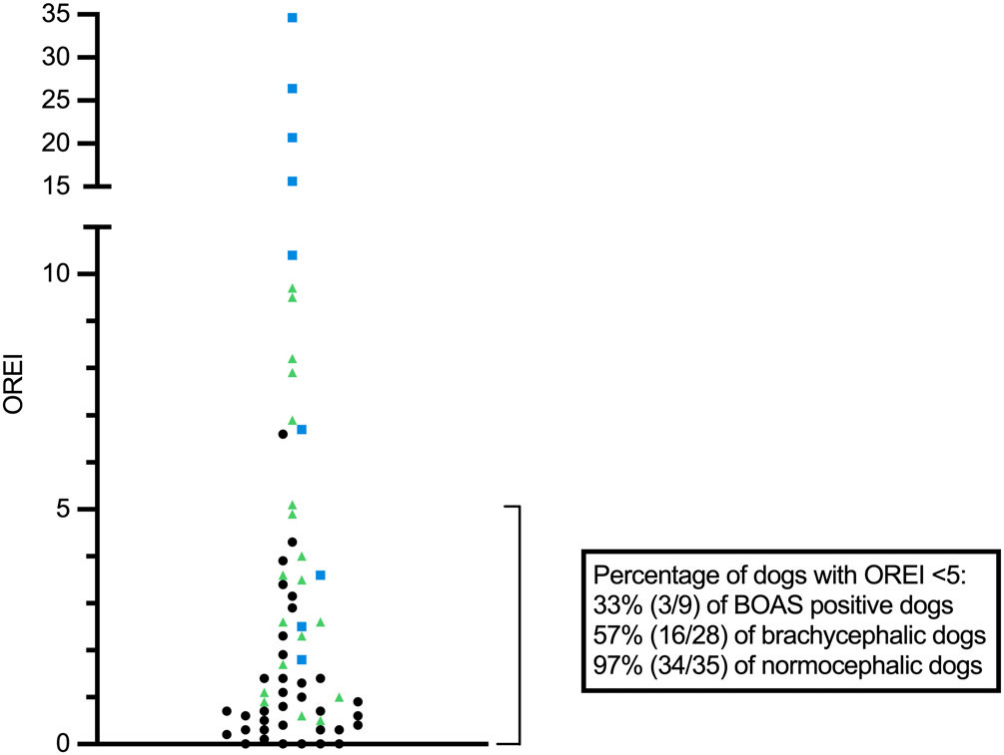
Scatter plot of obstructive respiratory event index (OREI) values in all dogs with sleep recordings. Black data points are normocephalic dogs and green triangles and blue squares are brachycephalic dogs. Blue squares denote dogs with moderate or severe brachycephalic obstructive airway syndrome signs (BOAS positive class).

OREI values are presented in Figure 5. In all dogs, the median OREI value was 1.4 (IQR 0.6‐4.0). For brachycephalic dogs, median OREI was 3.8 (IQR 1.9‐9.2) and for normocephalic dogs, 0.7 (IQR 0.3‐1.4). In all dogs with study visits, the median OREI value was 1.6 (IQR 0.6‐3.9). In brachycephalic dogs with study visits, the median was 3.6 (IQR 1.8‐10.4) and in normocephalic dogs with study visits, 0.9 (IQR 0.3‐2.3).

### 3.4 Risk factors for high OREI

In the whole study group, brachycephaly was the only significant risk factor (*P* < .001). In dogs with study visits, brachycephaly (*P* < .001) and excess weight, defined as a BCS of over 5/9, (*P* < .001) were identified as significant risk factors. The significant risk factors in brachycephalic dogs with study visits were BOAS‐positive class (BOAS severity grades 2 and 3; *P* = .03) and excess weight, defined as a BCS of over 5/9, (*P* = .009). The results from all univariable and multivariable analyses are presented in Table 2.

**TABLE 2:** Results from univariable and multivariable analyses of potential risk factors for high obstructive respiratory event index.

A. All dogs, n = 63			
	Univariate	Least square means	
	*P*‐value	Ratio of the geometric means (95% CI)	*P*‐value
Gender	.97		
Neuter status	.90		
Brachycephaly	<.001	5.62 (3.18‐9.94)	<.001
Age	.16		

B. Subgroups						
	All dogs with study visit, n = 46			Brachycephalic dogs with study visits, n = 19		
	Univariate	Multivariate		Univariate	Multivariate	
	*P*‐value	Ratio of the geometric means (95% CI)	*P*‐value	*P*‐value	Ratio of the geometric means (95% CI)	*P*‐value
Gender	.96					
Neuter status	.61					
Brachycephaly	<.001	5.77 (3.44‐9.67)	<.001			
Age	.32			.077		
Excess weight (BCS >5/9)	.007	3.37 (1.81‐6.68)	<.001	<.001	5.02 (1.59‐15.89)	.009
BOAS class				.005	2.49 (1.10‐5.61)	.03

Abbreviations: BCS, body condition score; BOAS, brachycephalic obstructive airway syndrome; CI, confidence interval.

## 4 DISCUSSION

This study explored our understanding of SDB in dogs using a portable neckband system, which measured the OREI value non‐invasively in the dogs' home environment. Brachycephaly and excess weight were risk factors for SDB, but not aging. In brachycephalic dogs, increasing severity of BOAS was identified as a risk factor. Signs of disturbed breathing during sleep were less common in dogs with a low OREI value of under 5.

In the questionnaire, the owners of 479 dogs evaluated whether their dog had signs of SDB. Sixty‐three dogs with a wide age range successfully underwent sleep recording. In addition to examining dogs with no owner‐perceived signs of SDB, we purposely selected dogs from certain breeds and with owner‐perceived signs of SDB, thus we expected to acquire more cases with SDB. We had an overrepresentation of Cavalier King Charles Spaniels, French Bulldogs and Labrador Retrievers. Brachycephalic dogs were emphasized as SDB has been reported in these.^1^, ^3^, ^4^ Labrador Retrievers were selected, as excess weight, a risk factor for OSA in humans,^34^ is prevalent in Labrador Retrievers.^35^ Thus, the selection process affects the results, and the prevalence of SDB in dogs cannot be estimated based on our results.

In the questionnaire results we found owner‐perceived indicators of sleep dysfunction to be more prevalent in dogs with a higher OREI value of over 5. Four dogs, all brachycephalic with very high OREI values of over 15, all had owner‐perceived signs of SDB during sleep—all snored and had apneic events.

Restless sleep occurred more in dogs with OREI values over 5 (39%, 5/13) compared to those with lower OREI values (26%, 13/50) as well as in brachycephalic dogs (43%, 12/28) compared to normocephalic dogs (17%, 6/35). Recurrent arousals and resulting daytime sleepiness are important diagnostic criteria for OSA in humans, as the frequent arousals resulting from obstructive apneas and hypopneas lead to unrefreshing sleep.^36^ Daytime sleepiness is included in the OSA questionnaire screening tools used to screen for OSA risk in humans.^29^ In dogs, the distinction between wake and sleep can be challenging to interpret because of the drowsiness sleep stage in dogs where their eyes can be closed or open and there a lack of motor activity.^37^ Additionally, sleep in dogs is polyphasic, that is, it occurs in smaller segments with wake periods in between, and although dogs are largely diurnal and sleep mainly during the nighttime, a considerable segment of sleep occurs during the day.^37^, ^38^, ^39^ Thus, daytime sleepiness can be difficult to interpret for the owner, but our results suggest that nighttime restlessness could be a sign of SDB in dogs. It must be noted, however, that other diseases, including osteoarthritis and canine cognitive dysfunction, can also cause restlessness.^40^, ^41^, ^42^, ^43^

Disturbances in sleeping position, that is, sleeping sitting up or with a toy in mouth, occurred more in both dogs with a high OREI value of over 5 compared to dogs with lower OREI, and in brachycephalic dogs compared to normocephalic dogs. Two normocephalic dogs, a Norwich Terrier and a Lancashire Heeler, rarely or sometimes slept sitting up. Attempting to sleep sitting up is reported in 24%^8^‐34%^15^ of brachycephalic dogs presenting for surgical treatment of BOAS, and not at all in normocephalic dogs.^8^ Sleeping with a toy or other item in their mouth is reported to occur seldomly in a study group of young brachycephalic dogs considered healthy by their owners, with 3% of owners (3/93) reporting such behavior.^13^ Similarly, in a group comprising young normocephalic and brachycephalic dogs, sleeping with a toy in mouth was described in 3.7% of all dogs.^38^ The effect of position of SDB in dogs has not been evaluated, but sleeping sitting up or with something in their mouth is widely assumed to reflect the dog's attempt to sleep in a position where the airflow is not compromised during sleep. SDB events are more prevalent during REM sleep,^2^ when the muscle tone of the upper airways is diminished, leaving the airways more susceptible to obstruction and collapse. Positional sleep apnea, where there is a 50% reduction in rate of apnea and hypopnea events during nonsupine sleep compared to supine sleep, is common in humans with mild OSA.^44^ However, the Lancashire Heeler that sleeps sitting up is notably normocephalic and had an OREI value of 1. Additionally, 1 normocephalic dog, a Kleinspitz, with an OREI value of 0, continuously slept with toy in mouth. It seems disturbances in sleeping position are not exclusively signs of SDB, but can occur because of other factors, including habit.

Apneic episodes witnessed by the owner, that is, not breathing for multiple seconds during sleep, were also described more in both dogs with a high OREI value of over 5 (46%, 6/13) compared to dogs with lower OREI (10%, 5/50), and in brachycephalic dogs (32%, 9/28) compared to normocephalic dogs (6%, 2/35). Previously, in brachycephalic dogs presented for surgical treatment with severe signs of BOAS, apneic episodes were present in 47% of dogs.^15^ Postoperatively, the described sleeping problems had largely resolved and only 3% had signs of SDB during sleep, highlighting the obstructive origin of the apneas in these dogs.^15^ In a group of both normocephalic and brachycephalic dogs aged 12 months, 0.5% of all dogs were reported to stop breathing sometimes during sleep.^38^ In our study a considerable proportion, 25% (7/28) of brachycephalic and 20% (7/35) of normocephalic dog owners, selected “I don't know” to the presence of apneic episodes. This uncertainty in choosing a more definite answer could be because of the owner not always being present to evaluate the dog's sleep. However, such ambivalence was not seen in response to the other questions. It could also reflect an unclear definition of an apneic episode in the questionnaire.

Snoring most of the time or continuously was common in dogs with high OREI value of over 5 and in brachycephalic dogs. Over 60% (9/13) of dogs with OREI value over 5 snored mostly or continuously compared to less than 20% (9/50) of dogs with OREI value under 5. The owner‐perceived proportion of snoring in brachycephalic dogs was 46% (13/28), which is less than previously published percentages ranging from 70% to 95%.^8^, ^13^, ^15^ In our study, owners reported snoring in 17% (6/35) of normocephalic dogs. Although preceding questionnaire studies are largely focused on the breathing and sleeping habits of brachycephalic dogs, loud breathing sounds are reported rarely in normocephalic dogs.^8^, ^38^ It is noteworthy that the owners did not always recognize signs of SDB. In some cases, we noticed a clear inconsistency between the objectively measured proportion of snoring and owner‐perceived signs, which makes snoring difficult for use as a risk factor for SDB. For example, an owner of a brachycephalic dog which snored for 60% of the duration of the sleep recording had answered “never snores” in the questionnaire. This was confirmed by interviewing the owner. Discrepancies between owner‐perceived health status and clinical findings are reported in brachycephalic dogs in both clinical^9^ and questionnaire^14^, ^45^ studies. The unrealistically good perceptions of brachycephalic dogs' health reported by owners can be because of cognitive dissonance where the owners, while being appreciative of these breed‐related issues, find them psychologically uncomfortable and reject the problems in their own pet.^14^ Additionally, owners might consider snoring normal for the brachycephalic breeds, and not regard it as a sign of conformational changes associated with brachycephaly.^45^

Obstructive apnea and hypopnea events during sleep can be quantified by measuring OREI in dogs. As was seen in our previous study,^4^ we noticed that brachycephaly was a risk factor for high OREI value. Brachycephalic dogs had more than 5 times higher OREI values than normocephalic dogs on average. In addition to brachycephaly, increasing severity of BOAS was identified as a risk factor for high OREI value. SDB events have been described previously by owners,^46^, ^47^ and apnea events have been objectively recorded in laboratory studies using polysomnography and whole‐body barometric plethysmography.^1^, ^3^ We recently objectively assessed SDB using the neckband system in the dog's home.^4^ The impact of increasing severity of BOAS grade on obstructive respiratory events during sleep has not been reported previously. However, it is well known that increasing severity of BOAS is associated with other consequences, such as poor exercise tolerance^13^, ^32^ and impaired respiratory function.^9^, ^48^ Furthermore, mandibular deficiencies and craniofacial disharmony, which includes increased anterior facial height and an inferiorly positioned hyoid, despite not being analogous to brachycephaly in dogs, can be present in humans with OSA.^19^, ^49^ In humans, brachycephaly is associated with OSA,^50^ although contradictory findings exist.^51^

A considerable proportion of brachycephalic dogs, 43% (12/28) had high OREI values of over 5, which is considered the threshold for OSA in adult man.^5^ Low OREI values of under 5 were most prevalent in normocephalic dogs (97%, 34/35), but over half of the brachycephalic dogs also had OREI values under 5. However, our study group does not depict a random sample representative of the whole population, as it consists of dogs recruited because of their breed and/or the presence or absence of owner‐perceived signs of SDB. As the participants were not selected from a population seeking treatment for signs of BOAS, it is possible that the most severe brachycephalic dogs are not included. Possibly bias also arises from the owners' potential greater incentive to participate if their dog had signs of SDB.

Regarding normocephalic dogs, it is noteworthy that 1 normocephalic dog, a Norwich Terrier, had an OREI value of over 5. Additionally, another Norwich Terrier had an OREI value of 4.3, which is high compared to median OREI values in normocephalic dogs. The owner of the latter Norwich Terrier reported signs of SDB, while the other did not. To our knowledge, this is the first time SDB is described in a normocephalic dog. The cause for upper airway obstruction in Norwich Terriers is Upper Airway Syndrome,^11^, ^12^ where the obstruction of airflow differs from BOAS and is associated with lymphoedema.^10^ The mutation associated with lymphoedema in Norwich Terriers is also observed in the Pomeranian and Mittelspitz, which are not included in our study population. The Norwich Terrier, although currently considered mesocephalic, is also suggested to be in transition to brachycephaly.^11^ This tendency, present also in some other normocephalic breeds, encompasses a true risk for impaired welfare in these dogs.

Interestingly, Chiari malformation, a structural defect of the skull resulting in herniation of the brain into the spinal canal, has been associated with CSA in humans.^52^ Chiari‐like malformation is prominent in many brachycephalic breeds, including the Cavalier King Charles Spaniel, where the defect is ubiquitous,^53^ but also in Pugs.^54^ However, central apnea or hypopnea events, excluding physiologic events related to sighs, did not occur in our study group. In a previous study,^3^ the resolution of apneas after extensive surgical procedures also supports obstructive, not central, apnea events in the Cavalier King Charles Spaniels studied.

Excess weight, defined as a BCS of over 5/9, was a risk factor for SDB. In contrast to earlier findings, excess weight being prevalent in Labrador Retrievers,^35^ only 1 out of 10 Labrador Retrievers was overweight. In people, obesity,^25^, ^34^, ^55^ particularly excess upper airway fat,^16^, ^20^ is a major risk factor for OSA. Although the association between obesity and SDB in dogs has not been investigated, obesity is a known risk factor for BOAS.^9^, ^48^, ^56^ Minute volume is decreased and both inspiratory and expiratory flow limited in obese brachycephalic dogs^48^ and tidal volume decreased also in obese normocephalic dogs.^57^ In humans, the exact mechanism by which obesity generates OSA is unclear, but it is presumed obesity leads to increased pharyngeal collapsibility because of impaired mechanical and neuromuscular effects.^58^, ^59^ Anatomic alterations contributing to increased mechanical resistance include decreased lung volume^60^ and narrowing of the upper airway because of fat deposition,^61^ as in brachycephalic dogs.^48^ Specifically, OSA severity increases with the severity of parapharyngeal fat deposition.^62^ Macroglossia contributes also to BOAS, and increased volume of tongue fat is reported in brachycephalic dogs.^63^, ^64^, ^65^

In humans with OSA, disturbances in neural response lead to the inactivation of compensatory neuromuscular responses, including dilatation and elongation of the upper airways, following airway obstruction.^58^ Increased connective tissue and abnormal morphology of muscle fibers are reported in the upper airway dilator muscles of English Bulldogs.^66^ Weight loss is an effective treatment form of OSA in humans,^18^, ^20^ and respiratory function is also improved by weight loss in healthy dogs.^67^

Aging was not identified as a risk factor for high OREI value. This can be impacted by the limited sample size and the several young brachycephalic dogs already considerably affected by BOAS. In humans, the prevalence of OSA increases with age.^23^, ^24^, ^25^ Signs of BOAS can also aggravate with age, as secondary changes, including tonsillar hypertrophy and laryngeal collapse, can worsen respiratory function.^68^ However, the assessment of BOAS severity remained relatively consistent in healthy young and middle‐aged brachycephalic dogs recently, when re‐evaluated at 2 to 3 years after an initial assessment at over 2 years of age.^69^

Neither gender nor neuter status were identified as risk factors for SDB. In people, the prevalence^27^, ^34^ and severity^70^ of OSA is greater in men. The gender difference decreases with age, with later onset of OSA in women,^23^ possibly because of postmenopausal hormonal changes.^27^ Such postmenopausal hormonal changes are not present in aging dogs, but to minimize variation in breathing during sleep caused by sex hormones during the estrous cycle, the sleep recordings were performed during anestrus in intact females.

This study has some limitations. The neckband device cannot be used in breeds with neck girth under 25 cm, and thus only dogs of suitable size could be recruited. Not all dogs attended a study visit, and thus comorbid conditions affecting breathing during sleep cannot be ruled out. Although BOAS severity grading has not been evaluated in brachycephalic breeds excluding the extremely brachycephalic English Bulldog, French Bulldog and Pug,^13^, ^32^, ^71^ we performed it similarly in the other brachycephalic breeds. Only 1 of the 9 BOAS positive dogs represented a breed other than the 3 aforementioned breeds. This individual was a Cavalier King Charles Spaniel. It is possible that the BOAS severity grading used here does not accurately quantify the respiratory signs seen in other brachycephalic breeds.

Brachycephaly affects the welfare of dogs in a plethora of ways, including disturbing sleep. Alongside brachycephaly, excess weight and increasing severity of BOAS predispose to SDB. To improve breathing during sleep, obesity should be avoided especially in brachycephalic dogs. Thresholds between normal and abnormal OREI values and severity grading of SDB need further research.

## CONFLICT OF INTEREST DECLARATION

Sari‐Leena Himanen has been a medical advisor for Nukute Ltd. and does not have a current financial relationship. No other authors declare a conflict of interest.

## OFF‐LABEL ANTIMICROBIAL DECLARATION

Authors declare no off‐label use of antimicrobials.

## INSTITUTIONAL ANIMAL CARE AND USE COMMITTEE (IACUC) OR OTHER APPROVAL DECLARATION

The study protocol was approved by the Committee of Experimental Animals of Southern Finland (ESAVI/10906/04.10.07/2017, ESAVI/34278/15.11.21/2021) and by the University of Helsinki Viikki Campus Research Ethics Committee (13/2020, 11/2021).

## HUMAN ETHICS APPROVAL DECLARATION

Authors declare human ethics approval was not needed for this study.

## Supporting information


**Data S1:** Questionnaire on sleep‐disturbed breathing and sleeping habits of Finnish dogs. Translated from Finnish.

**Data S2:** A picture of the neckband device and protective cover.
