# Supplementary material for: Evaluation of risk factors for sleep‐disordered breathing in dogs
Source: J Vet Intern Med. 2024 Feb 15;38(2):1135–45. doi: 10.1111/jvim.17019 (PMC10937515; doi:10.1111/jvim.17019)
Supplement: Supplementary file 1 — Data S1: Questionnaire on sleep‐disturbed breathing and sleeping habits of Finnish dogs. Translated from Finnish. [file JVIM-38-1135-s002.pdf]

Evaluation of risk factors for sleep-disordered breathing in dogs

Iida Niinikoski, Sari-Leena Himanen, Mirja Tenhunen, Mimma Aromaa, Liisa Lilja-Maula, Minna M. Rajamäki

Supporting Information S1. Questionnaire on sleep-disturbed breathing and sleeping habits of Finnish dogs. Translated from Finnish.

Owner name, number, email:

Name of dog:

Official registered name of dog:

Registration number of dog:

Breed: if mixed, write ”mixed”

Gender: Male / Male neutered / Female / Female sterilized

Date of birth: D-M-Y

Weight (kg):

Presence of other dogs in household: Y / N

- If yes, how many: 2 / 3 / 4 / 5 or more

Underlying diseases: Y / N / I don’t know

- If yes, which of the following:
  - Canine cognitive dysfunction / dementia
  - Epilepsy
  - Separation anxiety
  - Hypothyroidism
  - Pruritus: food allergy or atopic dermatitis
  - Osteoarthritis
  - Gastroesophageal reflux
  - Renal disease
  - Chronic respiratory disease, such as chronic bronchitis, canine idiopathic pulmonary fibrosis, or brachycephalic obstructive airway syndrome
  - Obsessive-compulsive disorder
  - Diabetes mellitus
  - Heart disease
  - Other, what:

Signs related to aforementioned disease are: occasional / constant

If occasional, when are they present?:

Is the dog currently on any medications: Y / N / I don’t know

If yes, which ones:

Betablockers, such as atenolol or sotalol

Antiepileptics, such as phenobarbital, potassium bromide, imepitoin

Separation anxiety medications, such as clomipramine

Bronchodilators, such as theophylline

Thyroid medicators, such as levothyroxine

Corticosteroids, such as prednisolone, methylprednisolone

Medication for urinary incontinence, such as phenylpropanolamine

Diuretics, such as furosemide, torasemide

Osteoarthritis injection bedinvetmab (Librela)

Other, what:

Has the dog been sick in the past 6 months: Y / N / I don’t know

If yes, what was the disease / condition and how long did it last? Please also report signs even in the absence of a distinct diagnosis or disease.

Has the dog had surgical operations on his/her upper airways (such as widening of nostrils, shortening of soft palate): Y / N / I don’t know

If yes, what and when:

Most common sleeping position:

On its side

On its back

Curled up in a loop

On its tummy

The usual sleeping place:

In the same bed with humans

On the floor in the same room with humans

In a different room to humans

Does your dog usually sleep

Calmly

Restlessly: for example changes places multiple times a night or walks around during the night

I don't know

During the last four weeks, has the dog:

| | Never | Rarely | Sometimes | Most of the time | Continuously | I don't know |
|---|---|---|---|---|---|---|
| .. snored in its sleep | | | | | | |
| .. changed sleeping places multiple times during the same night | | | | | | |
| .. slept sitting up | | | | | | |
| .. slept with a toy in its mouth | | | | | | |
| .. slept on its back | | | | | | |
| .. had loud, raspy breathing during sleep | | | | | | |
| .. slept with its head hanging off the bed | | | | | | |
| .. woken up from sleep gasping | | | | | | |
| .. had apneas during sleep, i.e. not breathing for multiple seconds during sleep | | | | | | |

You can clarify and expand on the dog's sleep and behaviors and their length:

Is the dog markedly sleep and/or tired during the day: Y / N / I don't know

Estimate how likely the dog would fall asleep during the following situations:

| | Would never fall asleep | Small likelihood to fall asleep | Moderate likelihood to fall asleep | Large likelihood to fall asleep |
|---|---|---|---|---|
| You watch television for 30 minutes and the dog is with you | | | | |
| You are at a friend's house for 30 minutes (no other pets or children present) | | | | |
| A guest comes to your house and you sit chatting for 30 minutes | | | | |
| You take the dog for vaccinations at the veterinary clinic and wait for 15 minutes in the waiting room before the appointment | | | | |

Does the dog wake up to sudden disturbances, such as loud sounds, immediately during the day when it's sleeping? Y / N / I don't know

During the last four weeks, has the dog:

| | Never | A few times | Multiple times | I don't know |
|---|---|---|---|---|
| .. twitched forcefully in its sleep (*tail wagging, moving its paws and small movements in the muscles around the eye is normal)* | | | | |
| .. howled, growled, or barked in its sleep (*minor whining is normal)* | | | | |
| .. suddenly got up in the middle of sleeping and attacked a nearby human or animal | | | | |

I allow the researchers to contact me for the second part of the study, the sleep recording: Y / N
