# Supplementary material for: Evaluation of risk factors for sleep‐disordered breathing in dogs
Source: J Vet Intern Med. 2024 Feb 15;38(2):1135–45. doi: 10.1111/jvim.17019 (PMC10937515; doi:10.1111/jvim.17019)

Evaluation of risk factors for sleep-disordered breathing in dogs

Iida Niinikoski, Sari-Leena Himanen, Mirja Tenhunen, Mimma Aromaa, Liisa Lilja-Maula, Minna M. Rajamäki

Supporting Information S2. A picture of the neckband device and protective cover.

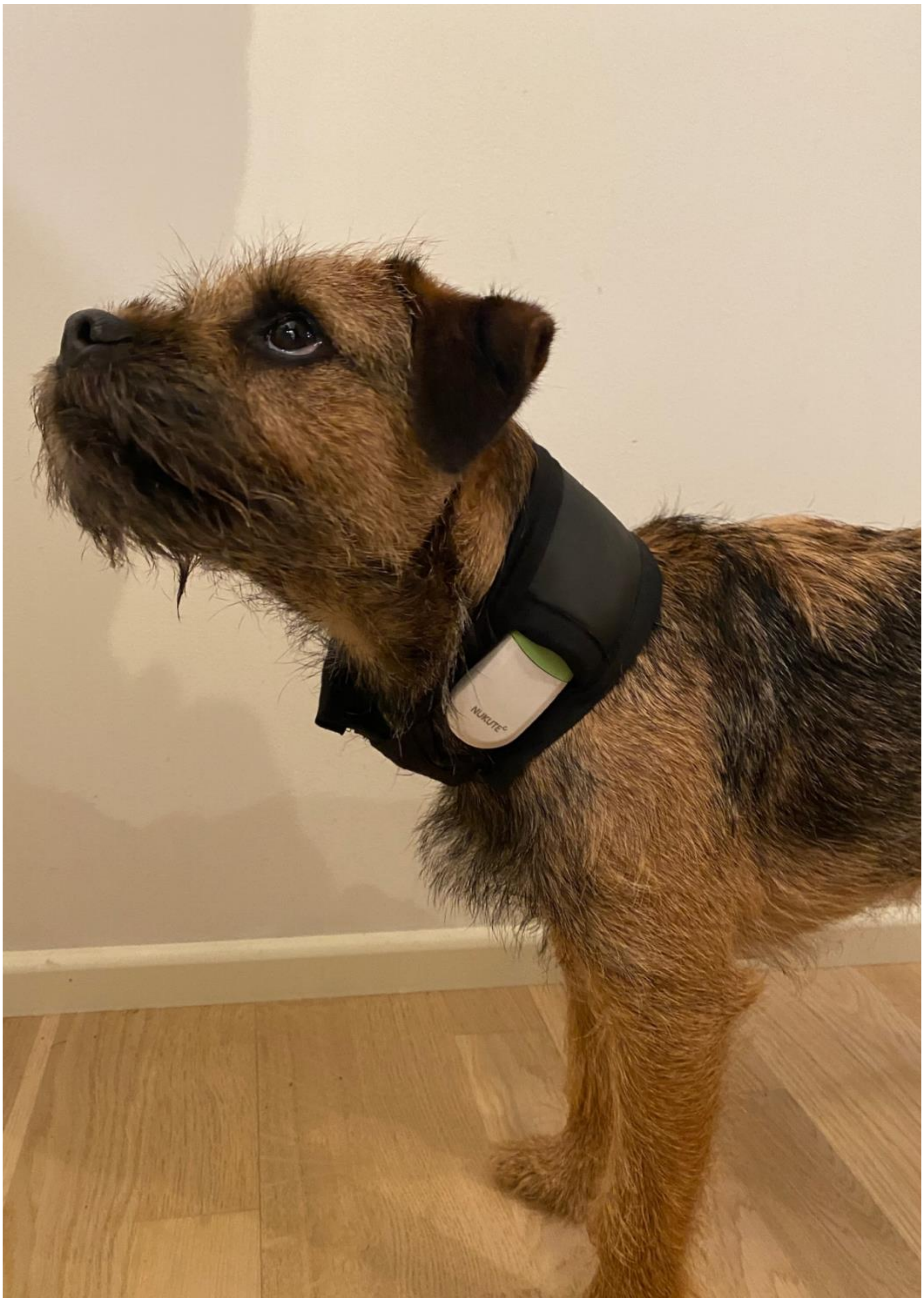

Supplement: Supplementary file 2 — Data S2: A picture of the neckband device and protective cover. [file JVIM-38-1135-s001.pdf]
